# Cervical cystic hygroma in adults: a case report

**DOI:** 10.1186/s13256-024-04624-x

**Published:** 2024-07-06

**Authors:** Shahnam Askarpour, Milad Arabi, Hossein Ghaedamini, Fardis Salmanpour

**Affiliations:** 1 Department of General Surgery, School of Medicine, Ahvaz Jundishapur University of Medical Sciences, Ahvaz, Islamic Republic of Iran; 2 Student Research Committee, School of Medicine, Ahvaz Jundishapur University of Medical Sciences, Ahvaz, Islamic Republic of Iran

**Keywords:** Cystic hygroma, Adult, Neck cyst, Iran, Case report

## Abstract

**Backgrounds:**

Manifestation of cystic hygroma in adulthood is very rare. The rarity of cystic hygroma in adults has caused problems in its diagnosis and management and few studies have reported cystic hygroma in adults.

**Case presentation:**

In this study, we reported a rare case with cervical cystic hygroma in adults. We report a 20-year-old Iranian male (Iranian ethnicity) with a diagnosis of right-side neck cystic hygroma and discuss the presentation, diagnosis, and clinical, radiological, and operative aspects of it.

**Conclusion:**

Cystic hygromas are a rare occurrence in adults. They are typically asymptomatic, rarely complicated, and can be mistaken for a cystic neck mass. This study showed that in our case, surgical resection may be a safe and effective treatment for cystic hygroma, with minimal risk of complications during the procedure.

## Background

Lymphatic malformation (cystic hygroma or lymphangioma) occurs as a result of sequestration or obstruction of developing lymph vessels in approximately 1 in 12,000 births [1].

Although the lesion can occur anywhere, the most common sites are in the posterior triangle of the neck, axilla, groin, and mediastinum [2].

The prevalence of this complication in adulthood is very rare. Adult presentation is related to congenital or acquired delay in lymphoid proliferation after trauma or previous respiratory infections [3].

Causes of cystic hygroma in adults include genetic conditions and environmental factors [4].

Symptoms and presentation consist of swelling and cystic growths, impact on adjacent organs and structures, and potential pain or discomfort [5].

Diagnosis is based on medical history and physical examination, imaging diagnostic tests [ultrasound, computed tomography (CT) scan, magnetic resonance imaging], biopsy, or fluid analysis [6].

Treatment options include surgical intervention and removal, medications or injections for symptom management, or physical therapy or rehabilitation post-surgery [7].

The rarity of cystic hygroma in adults has caused problems in its diagnosis and management, and few studies have reported cystic hygroma in adults [8–15]. In this study we reported a 20-year-old male with a diagnosis of left-side neck cystic hygroma.

## Case presentation

This research was approved by the ethics committee of the university with the ethics code IR.AJUMS.REC.1403.028. A 20-year-old Iranian male (Iranian ethnicity) came to the Surgery ward of Golestan Hospital of Ahwaz (southwest of Iran) with a complaint of painless swelling on the right side of his neck from 5 years ago.

At first, the patient was asymptomatic for 5 years after which he noticed a swelling in the neck; which has since gradually increased in size. The patient did not experience any difficulty swallowing or breathing. He had no medical, family, or psychosocial history. He also did not report any history of trauma or upper respiratory tract infection. He had not taken any action to address this issue.

**Fig. 1:**
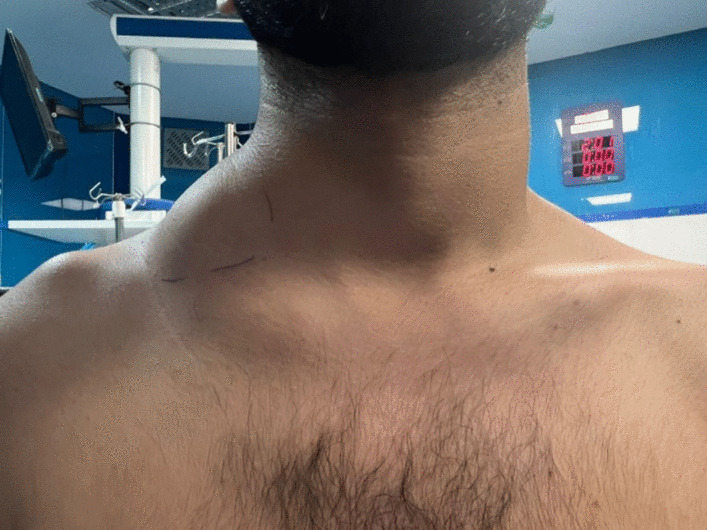
Male 20-year-old adult with cystic hygroma

During the examination, a significant swelling was observed on the right side of the neck, measuring 100 × 30 mm. The surface of the swelling was soft-lobulated and nontender. It was also fluctuant and extended into the right posterior triangle of the neck and right supraclavicular area (Fig. 1).

**Fig. 2:**
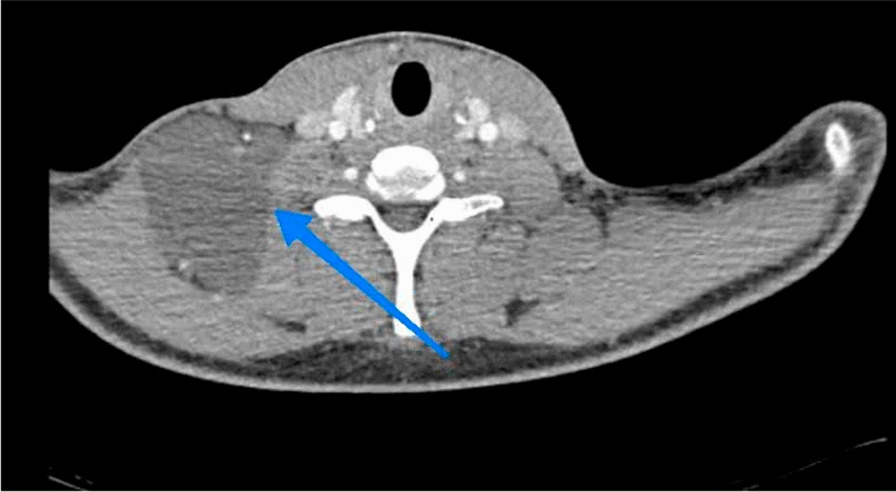
Transverse view of the neck computed tomography scan. The arrow shows a homogeneous cystic lesion in the right supraclavicular region, closely related to the vasculature of the head and neck

The spiral chest CT scan with and without contrast was normal but in the spiral neck CT scan with and without contrast reported a multilobulated hypodense area at supraclavicular regions measuring about 82 × 42 mm, suggestive of cystic hygroma. This is shown in Fig. 2.

**Fig. 3:**
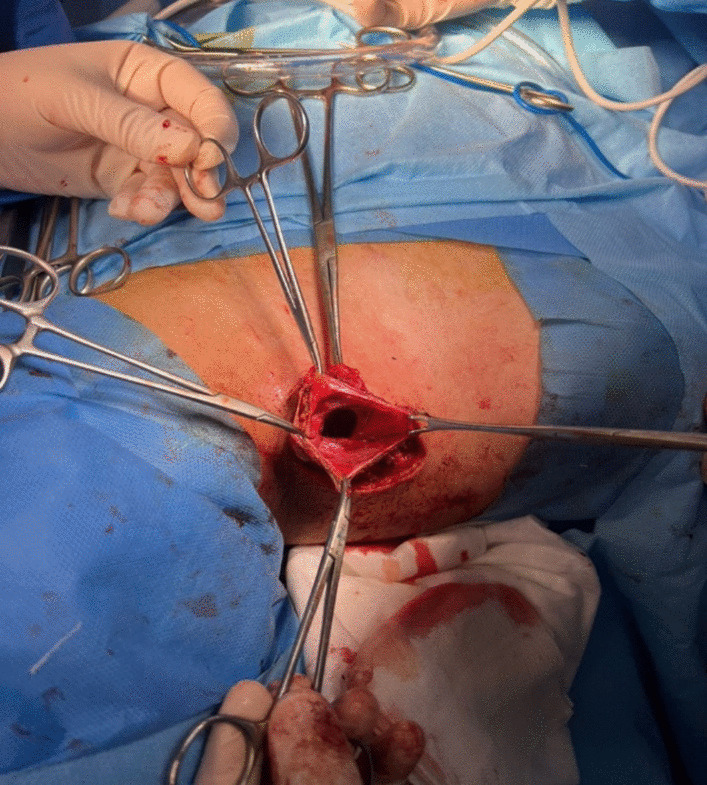
Intraoperative finding lobulated cystic lesion

**Fig. 4:**
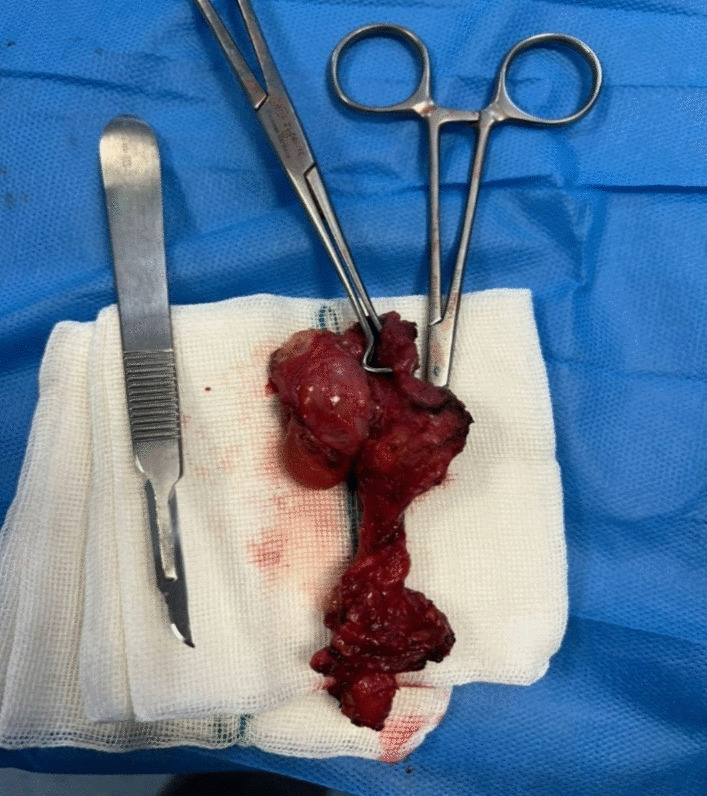
Cut section of the post cyst excision

**Fig. 5:**
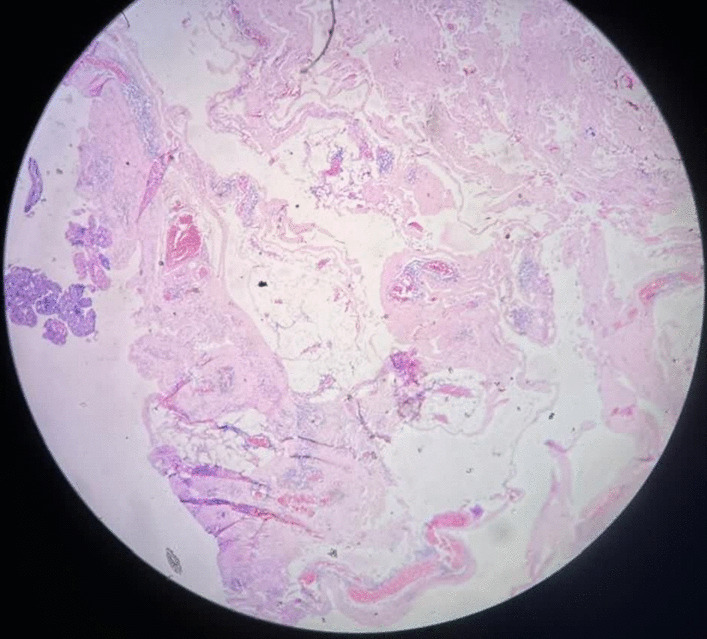
Histopathology examination of postoperative specimen shows multiple lymph spaces lined by lymphatic epithelium

The patient underwent radical cyst resection (Fig. 3). Because the cyst extended under the right clavicle (from the anterior view) to the posterior triangle of the neck, a classic cervical incision (posterior of the sternocleidomastoid muscle) was performed. The cyst was adherent to the surrounding tissues and the right internal jugular vein, that was released. The vessels and nerves of that area were saved (Fig. 4). We used talcum powder to prevent seroma formation. The patient visited the surgery clinic a week after being discharged, and his examination was normal. Additionally, he underwent an ultrasound the following month, which also showed normal results. The histopathological study supports the diagnosis of cavernous lymphangioma (cystic hygroma) (Fig. 5).

## Discussion

Manifestation of cystic hygroma in adulthood is very rare and its cause is controversial, but it is probably owing to delayed proliferation of congenital lymphatic malformation, trauma, and upper respiratory tract infection [8].

Our case had neck swelling that was painless, gradually increasing in size, asymptomatic, and without any compression symptoms, similar to the cases reported by Gow *et al*. [8], Kalsotra *et al*. [9], and Nasr *et al*. [10]. The most common presentation is an asymptomatic neck mass [2]. In some cases, it can lead to cosmetic disproportions, infection, feeding problems, and airway obstruction [5–7].

Cystic hygroma can be classified as either septated (multiloculated) or nonseptated single cavity (monoloculated) types. In our case, it was multiloculated, similar to the results of Nasr *et al*. [10] and McInerney *et al*. [11]. In cases of multiloculated cystic hygromas, surgery may be suitable, but minimizing surgical complications is necessary.

The results showed that the age of the patient in this case was 20 years old, placing him in the young age group. This finding is consistent with the results of Jayabal *et al*. [12] and Shrestha *et al*. [13]. Since cystic hygroma is most common in childhood, its frequency may decrease with increasing age, particularly in middle age and elderly groups.

The sex of our case was male, which is in line with the findings of Jayabal *et al*. [12] and Shrestha *et al*. [13], but contrary to the study by Colangeli *et al*. [14]. The results indicate that the sex ratio is equal among children [4], but further investigations are needed for adults.

The mass was located on the right side of the neck in our case, which is consistent with the findings of Nasr *et al*. [10], McInerney *et al*. [11], Jayabal *et al*. [12], Shrestha *et al*. [13], Colangeli *et al*. [14], and Piłkowski *et al*. [15]. It most commonly occurs in the head and neck region [8].

In the present study, CT scan and ultrasound were used as diagnostic tools. Jayabal *et al*. [11] utilized CT scan, Colangeli *et al*. [13] used ultrasound and magnetic resonance imaging (MRI), and McInerney *et al*. [14] used ultrasound, MRI, and CT scans.

MRI, CT scan, and ultrasonography are all helpful in diagnosing a cystic neck mass. MRI provides detailed images of soft tissues and can reveal the connection between the lesion and underlying structures [10]. Contrast can be used to differentiate between hemangiomas and lymphangiomas. CT scans carry a risk of radiation exposure and can make it challenging to distinguish the mass from surrounding tissue with similar properties [5]. The use of contrast can enhance visualization of the cyst wall and its proximity to nearby blood vessels. Ultrasonography is the least invasive option and can effectively show the relationship between the cyst and surrounding structures [8].

According to Morley *et al*. [16], Karakas *et al*. [17], and Nasr *et al*. [10], it is important to evaluate neck cystic hygroma using a combination of neck CT scan, ultrasonography, and MRI.

There are various treatment options for this condition, such as surgery, percutaneous drainage, sclerotherapy, laser therapy, and radiofrequency ablation.

In our case, we performed a complete surgical excision, as it is the most effective treatment for adult cystic hygroma. This approach is similar to the methods used by Nasr *et al*. [10], McInerney *et al*. [11], Jayabal *et al*. [12], Shrestha *et al*. [13], Colangeli *et al*. [14], and Piłkowski *et al*. [15]. Managing cystic hygroma can be challenging owing to its tendency to spread locally and reoccur after surgery.

Debate arose after the introduction of sclerotherapy, a procedure that involves injecting a sclerosant into the cyst to induce inflammation, thrombosis, and ablation.

So far, there has not been a randomized clinical trial comparing sclerotherapy with surgery. On the other hand, owing to the complex vascular structure and nerves in the neck, surgery is challenging and there is a possibility of recurrence.

The results indicated that our case underwent a 1-week and 1-month follow-up, with no evidence of recurrence or site infection, consistent with the findings of Nasr *et al*. [10], Jayabal *et al*. [11], Shrestha *et al*. [12], Colangeli *et al*. [13], McInerney *et al*. [14], and Piłkowski *et al*. [15]. However, the recurrence rate following incomplete excision of cystic hygroma is 10–50%, while removing only a portion of the lymphatic malformation results in a much higher rate of 88% [2]. It is crucial to discuss the risk of recurrence with the patient before the operation as morbidity can lead to cosmetic disfigurement and affect critical structures, such as nerves, blood vessels, lymphatics, and the airway.

Altogether cystic hygromas are a rare occurrence in adults. They are typically asymptomatic, rarely complicated, and can be mistaken for a cystic neck mass. Surgical resection may be a safe and effective treatment for cystic hygroma.

## Conclusion

Cystic hygromas are a rare occurrence in adults. They are typically asymptomatic, rarely complicated, and can be mistaken for a cystic neck mass. This study showed that in our case, surgical resection may be a safe and effective treatment for cystic hygroma, with minimal risk of complications during the procedure.

## Data Availability

The datasets generated during and/or analyzed during the current study are available from the corresponding author upon reasonable request. The materials used in this study are available commercially or can be obtained from the authors upon request.
